# Supplementary material for: Analysis of Newcastle disease virus prevalence in wild birds reveals interhost transmission of genotype VI strains
Source: Microbiol Spectr. 2024 Nov 8;12(12):e00816-24. doi: 10.1128/spectrum.00816-24 (PMC11619417; doi:10.1128/spectrum.00816-24)
Supplement: Supplemental material — Tables S1 to S5. [file spectrum.00816-24-s0001.docx]

**Supplementary Table** **1** Sample and isolation details

| Species (common name) | Species (scientific name) | Order | Habitat | Numbers of birds | NDV positive |
| --- | --- | --- | --- | --- | --- |
| Francolin | *Francolinus pintadeanus* | Galliformes | Resident | 749 | 6 |
| Turtle dove | *Streptopelia orientalis* | Columbiformes | Resident | 293 | 10 |
| Spotted dove | *Spilopelia chinensis* | Columbiformes | Resident | 185 | 5 |
| Pigeon | *Columba livia* | Columbiformes | Caged | 1316 | 9 |
| Pheasant | *Ithaginis cruentus* | Ithaginis cruentus | Resident | 465 | 3 |
| Sparrow | *Passer montanus* | Passeriformes | Resident | 10 | 0 |
| Quail | ***Coturnix coturnix*** | Galloanserae | Resident | 515 | 3 |
| Eurasian wigeon | ***Mareca penelope*** | Anseriformes | Migratory | 5 | 0 |
| Mallard | *Anas platyrhynchos* | Anseriformes | Migratory | 3 | 0 |
| Greater coucal | *Centropus sinensis* | Cuculiformes | Resident | 2 | 0 |
| Corn crake | *Crex crex* | Gruiformes | Resident | 2 | 0 |
| Jungle fowl | *Gallus gallus* | Galliformes | Resident | 2 | 0 |
| Pheasant-tailed jacana | *Hydrophasianus chirurgus* | Charadriiformes | Migratory | 2 | 0 |
| Pigeon(diseased) | *Columba livia* | Columbiformes | Caged | 20 | 3 |
| Total |  |  |  | 3469 | 39 |

**Supplementary Table** **2** Accession number, pathogenicity test result, genotype and F protein cleave sites characteristic of NDV strains

| Strain | Accession number | Genotype | MDT | ICPI | Cleave sites of F protein |
| --- | --- | --- | --- | --- | --- |
| Pigeon/China/GX03/2018 | OR099828 | Class Ⅰ genotype 1.1.2 | - | - | 112ERQGR↓L117 |
| Francolin/China/GX05/2019 | OR099829 | Class Ⅰ genotype 1.1.2 | - | - | 112ERQER↓L117 |
| Francolin/China/GX07/2019 | OR085374 | Class Ⅰ genotype 1.1.2 | - | - | 112ERQER↓L117 |
| Turtle dove/China/GX01/2018 | OR099824 | class Ⅱ genotype Ⅰ | - | - | 112GKQGR↓L117 |
| Francolin/China/GX03/2019 | OR085372 | class Ⅱ genotype Ⅰ | - | - | 112GKQGR↓L117 |
| Francolin/China/GX06/2019 | OR099827 | class Ⅱ genotype Ⅰ | - | - | 112GKQGR↓L117 |
| Pigeon/China/GX02/2019 | OR099825 | class Ⅱ genotype Ⅰ | - | - | 112GKQGR↓L117 |
| Pigeon/China/GX03/2019 | OR085371 | class Ⅱ genotype Ⅰ | - | - | 112GKQGR↓L117 |
| Pigeon/China/GX09/2019 | OR085375 | class Ⅱ genotype Ⅰ | - | - | 112GKQGR↓L117 |
| Pheasant/China/GX04/2018 | OR099826 | class Ⅱ genotype Ⅱ | - | - | 112GKQGR↓L117 |
| Pigeon/China/GXD31/2016 | MZ405135 | class Ⅱ genotype Ⅵ2.1.1.2.1 | 75 | 0.95 | 112RRQKR↓F117 |
| Pigeon/China/GXD27/2018 | MZ306219 | class Ⅱ genotype Ⅵ2.1.1.2.1 | 70 | 1.01 | 112RRQKR↓F117 |
| Quail/China/GX08/2019 | MZ620697 | class Ⅱ genotype Ⅵ2.1.1.2.1 | 120.8 | 1.05 | 112RRQKR↓F117 |
| Pigeon/China/GX12/2019 | MZ620689 | class Ⅱ genotype Ⅵ2.1.1.2.1 | 100 | 0.875 | 112RRQKR↓F117 |
| Pigeon/China/GXD28/2020 | MZ405137 | class Ⅱ genotype Ⅵ2.1.1.2.2 | 94.8 | 1.1 | 112RRQKR↓F117 |
| Pigeon/China/GX04/2016 | MZ395248 | class Ⅱ genotype Ⅵ2.1.1.2.2 | 104 | 0.75 | 112RRQKR↓F117 |
| Turtle dove/China/GX06/2016 | MZ395250 | class Ⅱ genotype Ⅵ2.1.1.2.2 | 72 | 0.625 | 112RRQKR↓F117 |
| Turtle dove/China/GX08/2016 | MZ400779 | class Ⅱ genotype Ⅵ2.1.1.2.2 | 68 | 1.1 | 112RRQKR↓F117 |
| Turtle dove/China/GX09/2016 | MZ400780 | class Ⅱ genotype Ⅵ2.1.1.2.2 | 85.4 | 0.94 | 112RRQKR↓F117 |
| Pigeon/China/GX03/2017 | MZ363634 | class Ⅱ genotype Ⅵ2.1.1.2.2 | 111.4 | 0.95 | 112RRQKR↓F117 |
| Turtle dove/China/GX05/2017 | MZ395249 | class Ⅱ genotype Ⅵ2.1.1.2.2 | 116 | 0.375 | 112RRQKR↓F117 |
| Pigeon/China/GX07/2017 | MZ395251 | class Ⅱ genotype Ⅵ2.1.1.2.2 | 92 | 0.76 | 112RRQKR↓F117 |
| Spotted dove/China/GX08/2017 | MZ620686 | class Ⅱ genotype Ⅵ2.1.1.2.2 | 100 | 0.75 | 112RRQKR↓F117 |
| Spotted dove/China/GX09/2017 | MZ620687 | class Ⅱ genotype Ⅵ2.1.1.2.2 | 110 | 0.375 | 112RRQKR↓F117 |
| Turtle dove/China/GX10/2018 | MZ620688 | class Ⅱ genotype Ⅵ2.1.1.2.2 | 96 | 1.05 | 112RRQKR↓F117 |
| Turtle dove/China/GX11/2018 | MZ306222 | class Ⅱ genotype Ⅵ2.1.1.2.2 | 58.4 | 1.19 | 112RRQKR↓F117 |
| Spotted dove/China/GX15/2018 | MZ620690 | class Ⅱ genotype Ⅵ2.1.1.2.2 | 99 | 0.425 | 112RRQKR↓F117 |
| Turtle dove/China/GX16/2018 | MZ620693 | class Ⅱ genotype Ⅵ2.1.1.2.2 | 100 | 0.275 | 112RRQKR↓F117 |
| Turtle dove/China/GX17/2018 | MZ620694 | class Ⅱ genotype Ⅵ2.1.1.2.2 | 81.4 | 0.35 | 112RRQKR↓F117 |
| Pigeon/China/GX01/2019 | OR085370 | class Ⅱ genotype Ⅵ2.1.1.2.2 | 69.2 | 0.875 | 112RRQKR↓F117 |
| Pheasant/China/GX04/2019 | MZ620695 | class Ⅱ genotype Ⅵ2.1.1.2.2 | 88.4 | 1.01 | 112RRQKR↓F117 |
| Quail/China/GX05/2019 | OR085373 | class Ⅱ genotype Ⅵ2.1.1.2.2 | 72.4 | 1.075 | 112RRQKR↓F117 |
| Spotted dove/China/GX06/2019 | MZ620696 | class Ⅱ genotype Ⅵ2.1.1.2.2 | 81.6 | 0.81 | 112RRQKR↓F117 |
| Spotted dove/China/GX07/2019 | MZ405131 | class Ⅱ genotype Ⅵ2.1.1.2.2 | 101.6 | 0.775 | 112RRQKR↓F117 |
| Turtle dove/China/GX11/2019 | MZ620698 | class Ⅱ genotype Ⅵ2.1.1.2.2 | 70 | 0.375 | 112RRQKR↓F117 |
| Pheasant/China/GX13/2019 | MZ620691 | class Ⅱ genotype Ⅵ2.1.1.2.2 | 93 | 0.8 | 112RRQKR↓F117 |
| Quail/China/GX14/2019 | MZ620692 | class Ⅱ genotype Ⅵ2.1.1.2.2 | 79 | 0.825 | 112RRQKR↓F117 |
| Francolin/China/GX01/2017 | MZ306226 | class Ⅱ genotype Ⅻ | 58 | 1.613 | 112RRQKR↓F117 |
| Francolin/China/GX02/2017 | MZ306225 | class Ⅱ genotype Ⅻ | 57 | 1.65 | 112RRQKR↓F117 |

**Note:** According to OIE protocol, an intracerebral pathogenicity index (ICPI) is greater than 0.7 to be classified as virulent strain.

**Supplementary Table** **3** Reference strains information of Class Ⅰ NDVs

| Accession number | Strain name | Accession number | Strain name |
| --- | --- | --- | --- |
| AB858995 | J17/13 | KF361507 | mallard/Jilin/2011 |
| AB858996 | J72/13 | KF444680 | MUSW/1180 |
| AB858997 | J55/13 | KJ499462 | sw/CH/LHLJ/120608 |
| AB858998 | J77/13 | KJ607171 | pf/CH/LHLJ/131047 |
| AB858999 | J80/13 | KM669995 | SD21/13 |
| AB859000 | J36/13 | KM669996 | SD22/13 |
| AB859001 | J70/13 | KM885150 | Du/CH/LZJ/068/2011 |
| AB859002 | J2/13 | KM885151 | Du/CH/LJS/215/2011 |
| AB871656 | APMV/1/duck/Tottori/453/2009 | KM885152 | Du/CH/LZJ/067/2011 |
| AB871657 | APMV/1/duck/Tottori/481/2009 | KM885153 | Du/CH/LJS/016/2011 |
| AY034801 | Newcastle/disease/virus/strain/Fin/97 | KM885155 | Du/CH/LGX/298/2011 |
| AY626266 | duck/US/119535/1/2001 | KM885156 | Du/CH/LFJ/048/2011 |
| AY626267 | duck/US/154979/1/2001 | KM885157 | Du/CH/LGD/364/2011 |
| AY626268 | chicken/US/101250/2/2001 | KM885159 | Du/CH/LFJ/104/2011 |
| DQ097393 | DE/R49/99 | KM885160 | Du/CH/LFJ/027/2011 |
| EF564813 | green/winged/teal/US/AK/176/1998 | KM885161 | Du/CH/LHuB/085/2013 |
| EF564819 | red/knot/US/DE/2026/2000 | KM885162 | Du/CH/LGX/280/2013 |
| EF564820 | mallard/US/MD/04/125/2004 | KM885163 | Du/CH/LFJ/073/2013 |
| EF612277 | Northern/Pintail/US/AK/196/1998 | KM885168 | Du/CH/LGD/358/2011 |
| EU493451 | APMV/1/Teal/Finland/12104/06 | KT223818 | NDV/pigeon/China/Qinghai/01/2014 |
| EU493454 | APMV/1/Pochard/Finland/13193/06 | KT381586 | Duck/Guangdong/YF827/2014 |
| FJ487637 | NDV08/004 | KT381587 | Pigeon/Guangdong/YF1/2014 |
| FJ597580 | D/ZJ/1/02 | KT381588 | Chicken/Guangdong/GM3/2013 |
| FJ597581 | D/ZJ/3/02 | KT381589 | Chicken/Guangdong/GM307/2014 |
| FJ597582 | D/JS/1/07 | KT892746 | chicken/CH/JL/CC02/2015 |
| FJ597583 | D/JS/2/07 | KT892748 | chicken/CH/JL/CC03/2014 |
| FJ597584 | D/JS/16/05 | KU366517 | APMV/1/shelduck/Chokpak/5717/2013 |
| FJ597585 | D/AH/10/06 | KX602322 | Sheldrake/duck/China/Guizhou/01/2016 |
| FJ597586 | D/FJ/2/02 | KX602323 | Sheldrake/duck/China/Guizhou/02/2016 |
| FJ597587 | D/JS/9/06 | KX857692 | Ruddy/Turnstone/USA/NJ/AI13/2469/2013 |
| FJ597588 | D/JS/17/05 | KX857693 | mallard/USA/MN/AI13/3303/2013 |
| FJ597589 | D/JS/18/05 | KX857694 | Blue/winged/Teal/USA/TX/AI13/3994/2013 |
| FJ597600 | D/ZJ/30/05 | KX857695 | Blue/winged/Teal/USA/TX/AI13/4042/2013 |
| FJ597601 | D/ZJ/31/05 | KX857696 | Blue/winged/Teal/USA/TX/AI14/2572/2014 |
| HQ398788 | NDV09/014 | KY776595 | LTL130830 |
| GQ245777 | XZ/35/07/Ch | MF100727 | C/GD/GZ/148/2016 |
| HQ008337 | JS10 | MF100728 | C/GD/GZ/150/2016 |
| HQ398777 | NDV08/037 | MF100729 | C/GD/GZ/191/216 |
| HQ398778 | NDV08/038 | MF100730 | C/GD/HZ/390/2016 |
| HQ398780 | NDV08/044 | MF100731 | C/GD/HZ/398/2016 |
| HQ398794 | NDV09/020 | MF100732 | C/GD/HZ/404/2016 |
| HQ398795 | NDV09/022 | MF100733 | C/GD/DG/410/2016 |
| HQ398799 | NDV09/031 | MF100734 | C/GD/YF/445/2016 |
| HQ398801 | NDV09/042 | MF100735 | C/GD/YF/607/2016 |
| HQ398802 | NDV09/043 | MF100736 | C/GD/YF/609/2016 |
| HQ398803 | NDV09/044 | MF100737 | C/GD/DG/618/2016 |
| HQ398805 | NDV09/047 | MF100738 | C/GD/DG/698/2016 |
| HQ398807 | NDV09/049 | MF100739 | C/GD/DG/707/2016 |
| HQ398809 | NDV09/052 | MF100741 | C/GD/GZ/999/2016 |
| HQ398810 | NDV09/053 | MF100742 | C/GD/HZ/140/2016 |
| HQ398812 | NDV09/055 | MF100743 | D/GD/HZ/154/2016 |
| HQ398813 | NDV09/056 | MF100744 | D/GD/YF/193/2016 |
| HQ398814 | NDV09/057 | MF100745 | D/GD/YF/458/2016 |
| HQ412767 | NDV08/046 | MF100746 | D/GD/YF/706/2016 |
| HQ997378 | NDV10/005 | MF100747 | D/GD/DG/822/2016 |
| HQ997379 | NDV10/006 | MF100748 | D/GD/GZ/825/2016 |
| HQ997380 | NDV10/011 | MF100749 | E/GD/DG/12/2016 |
| HQ997382 | NDV10/059 | MF100750 | E/GD/GZ/402/2016 |
| HQ997383 | NDV10/060 | MF100751 | E/GD/HZ/406/2016 |
| HQ997387 | NDV10/064 | MF100752 | E/GD/YF/408/2016 |
| HQ997388 | NDV10/067 | MF100753 | E/GD/YF/580/2016 |
| HQ997389 | NDV10/069 | MF100754 | E/GD/DG/168/2016 |
| HQ997390 | NDV10/072 | MF100755 | G/GD/YF/392/2016 |
| HQ997391 | NDV10/079 | MF100756 | G/GD/YF/401/2016 |
| HQ997392 | NDV10/083 | MF100757 | G/GD/HZ/460/2016 |
| HQ997393 | NDV10/084 | MF100758 | G/GD/DG/571/2016 |
| HQ997394 | NDV10/085 | MF100759 | P/GD/GZ/178/2016 |
| HQ997397 | NDV10/089 | MF100760 | P/GD/DG/466/2016 |
| HQ997398 | NDV10/090 | MF100761 | P/GD/HZ/599/2016 |
| JF893453 | JX07 | MF100763 | D/GD/GZ/817/2016 |
| JN688862 | Ch/JS/09/03 | MH274991 | CK/GX/26/15 |
| JN688864 | D/AH/10/02 | MH274992 | CK/GX/65/15 |
| JN688865 | G/JS/09/08 | MH289856 | NDV/Environment/CN/HN/35S/2016 |
| JX844030 | NDV/mallard/Finland/9147/2010 | MH289857 | NDV/Goose/CN/HN/20S/2016 |
| KC503412 | Anas/sp/Japan/10UO0343/2010 | MH289858 | NDV/Chicken/CN/HN/50W1/2016 |
| KC503468 | northern/pintail/AK/44493/828/2009 | MH289861 | NDV/Duck/CN/HN/67B2/2016 |
| KC503470 | northern/pintail/AK/44493/865/2009 | MH289862 | NDV/Chicken/CN/JX/16I2/2016 |
| KC503472 | northern/pintail/AK/44494/659/2009 | MH289864 | NDV/Chicken/CN/HN/94N/2016 |
| KC503475 | American/green/winged/teal/AK/44494/794/2009 | MH289867 | NDV/Chicken/CN/HN/54L2/2017 |
| KC503483 | slaty/backed/gull/Russia/Nikita543FFNK15/2007 | MH289868 | NDV/Chicken/CN/JX/28A2/2016 |
| MH289869 | NDV/Anser/fabalis/CN/HN/F2/95/1/2016 | MH289917 | NDV/Environment/CN/JX/13M/2016 |
| MH289870 | NDV/Chicken/CN/HN/6T/2016 | MH289918 | NDV/Environment/CN/JX/37U/2016 |
| MH289873 | NDV/Environment/CN/JX/192X/2016 | MH289919 | NDV/Duck/CN/JX/Y82/2016 |
| MH289874 | NDV/Environment/CN/HN/74W/2016 | MH289920 | NDV/Environment/CN/JX/Y98/2016 |
| MH289875 | NDV/Chicken/CN/HN/15W1/2016 | MH289921 | NDV/Duck/CN/JX/57A2/2016 |
| MH289876 | NDV/Environment/CN/HN/54S/2016 | MH289922 | NDV/Duck/CN/HN/K86/2017 |
| MH289878 | NDV/Chicken/CN/HN/73V1/2016 | MH289930 | NDV/Chicken/CN/HN/K200/2017 |
| MH289879 | NDV/Chicken/CN/HN/Y10/2016 | MH289932 | NDV/Chicken/CN/HN/6W1/2016 |
| MH289881 | NDV/Environment/CN/HN/78T/2016 | MH289944 | NDV/Duck/CN/JX/69H2/2016 |
| MH289882 | NDV/Chicken/CN/HN/91N/2016 | MH289946 | NDV/Chicken/CN/JX/19H2/2016 |
| MH289885 | NDV/Environment/CN/HN/49S/2016 | MH289948 | NDV/Duck/CN/JX/57C2/2016 |
| MH289890 | NDV/Environment/CN/HN/89S/2016 | MH289949 | NDV/Environment/CN/JX/Y94/2016 |
| MH289893 | NDV/Environment/CN/HN/79S/2016 | MH289950 | NDV/Environment/CN/JX/Y95/2016 |
| MH289894 | NDV/Chicken/CN/HN/Y15/2016 | MH289953 | NDV/Chicken/CN/HN/41M2/2017 |
| MH289898 | NDV/Environment/CN/HN/K67/2017 | MH289959 | NDV/Chicken/CN/HN/53L2/2017 |
| MH289899 | NDV/Environment/CN/JX/47X/2016 | MH289960 | NDV/Duck/CN/JX/56I2/2016 |
| MH289901 | NDV/Environment/CN/JX/108G2/2017 | MH289963 | NDV/Chicken/CN/JX/50I2/2016 |
| MH289905 | NDV/Chicken/CN/JX/82G2/2017 | MH289966 | NDV/Environment/CN/JX/55U/2016 |
| MH289908 | NDV/Chicken/CN/JX/30F2/2017 | MH289970 | NDV/Environment/CN/JX/43X/2016 |
| MH289910 | NDV/Chicken/CN/HN/19B2/2016 | MK122776 | Chicken/China/SC/PT3/2016 |
| MH289913 | NDV/Wild/birds/CN/HB/F2B6/2/2017 | MH289914 | NDV/Environment/CN/JX/57U/2016 |

**Supplementary Table** **4** Reference strains information of Class Ⅱ NDVs

| Accession number | Strain name | Accession number | Strain name |
| --- | --- | --- | --- |
| AY935490 | 2/1334 | FJ751918 | QH1 |
| AY935495 | 99/868/hi | JX012096 | AF2240 |
| M24693 | Queensland | AF458009 | FJ/1 |
| EF564816 | NJ/A/101/1383 | FJ436303 | ZJ/1 |
| GQ918280 | BHG/Sweden/94 | FJ436302 | F48E8 |
| KX352834 | Tyva/14 | FJ705468 | TX/130 |
| AB465607 | Ishi | KX857716 | ndv42/AI09/4117 |
| KC503476 | AK/44500/136 | FJ705466 | 99/376 |
| KC503479 | Nikita/530/FFNK2 | KX857721 | MN/AI10/3434 |
| AY965079 | FarEast/2713 | HQ266602 | MG/725 |
| HG326605 | NIE08/121 | JX518882 | MGMNJ |
| KC503453 | AK/44493/716 | JX518884 | MGS1595T |
| AF077761 | Lasota | KU594615 | Apurimac/50009 |
| GU978777 | TX/GB | KU594616 | Lurin/40871 |
| JN872151 | Hitchner/B1 | KU594618 | Arequipa/VFAR/81 |
| EF201805 | Mukteswar | JN627504 | GD/12 |
| GU182327 | SPVC/Karachi/1 | JN627507 | GD/1003 |
| MH996904 | Novo/Selo/1161 | MF278927 | FS/SS/292 |
| AY741404 | Herts | JN942034 | 45445/3 |
| MH996900 | Plovdiv/1153 | JN942043 | 47385/11 |
| JN872189 | Coast/8278 | MF409241 | Chiwoko |
| JN872194 | 498109/15 | GU182323 | SPVC/Karachi/43 |
| JN942027 | 95066/9 | GU182331 | SPVC/Karachi/33/ |
| EU518682 | Distrito/Federal/462 | KF113338 | University/Diagnostic/Lab/12 |
| EU518684 | Estado/de/Mexico/466 | KM056349 | ndv42/gopalpura/4 |
| JQ697744 | NC04/635 | KT734767 | Polashbari |
| JX901367 | PA/810 | KX372707 | Nagpur/3 |
| JX901351 | NJ/721 | JQ267579 | EMM/7 |
| MG018211 | TX/1185/kidney/26981/3/A | JQ267584 | EMM/2 |
| JX094510 | sms12 | JQ267585 | EMM/1 |
| JX901110 | 248/ | HF969205 | NIE09/2071 |
| JX486553 | LHLJ/110813 | JN872165 | VIR/1377/7 |
| KT163262 | SH/167 | JQ039386 | VRD08/36 |
| JX901124 | 11/09620 | HF969187 | NIE08/453 |
| MG840654.1 | Ningxia/2068 | HF969210 | NIE10/139 |
| AF109885 | GB1168 | KY171990 | KD/TW/03T/N45/720 |
| FJ410145 | NY | JX915242 | 28138/4 |
| FJ865434 | S/1 | JX915243 | Queretaro/452/1947 |
| FJ480825 | PG/JS/1 | JX186997 | 867 |
| JX244794 | 100 | HF969176 | NIE10/310 |
| KJ607163 | LJS/1 | HF969191 | NIE08/2042 |
| JN872180 | TX/209682 | HF969194 | NIE08/2199 |
| JN872182 | 12339 | FJ772455 | 1532/14 |
| JX901312 | 101 | JF966389 | ML038 |
| HG326604 | NIE09/1898 | JX518885 | ML57051T |
| JX518532 | B2/Isiolo | HF969218 | CIV08/42 |
| HG424627 | NIE13/92 | HG326600 | CIV08/32 |
| EF589133 | 98/Guizhou | JX518886 | ML57072T |
| EF579733 | Shandong/Pyan | FJ705456 | MN/92/40140 |
| AB853927 | Ibaraki/SG106 | JN942024 | WI/272409 |
| KC542905 | Liaoning/1/2009 | KC433530 | FL/41105 |
| KX268351 | Behshahr | AB853928 | Ibaraki/SM87 |
| AY028995 | A7 | AF458016 | ZhJ/2 |
| GQ338309 | 18 | KY042142 | 88/M |
| DQ227246 | Jiangsu/JS02 | KC205479 | ETHMG1C |
| MF622047 | RBWW/3 | JN638234 | 11RS98/102VIR |
| KU862293 | Karachi/AW/1 | KU377533 | 10VIR7155 |
| HQ697254 | Banjarmasin/10 | KU377535 | 12VIR1876/1 |
| KY747479 | 5620 | KU862298 | Lahore/AW/2 |
| JN986837 | 152608/ancestral | KY042135 | 22A |
| AY734534 | Trenque/Lauquen | KY042141 | Jallo/Lahore/221B |
| AY935490 | 2/1334 | JF824032 | Vladimir/687 |
| AY935495 | 99/868/hi | KY042136 | Lahore/125 |

**Supplementary Table** **5** Reference strains information of Class Ⅱ genotypeⅥ.2.1.1.2.1 and Ⅵ.2.1.1.2.2 NDVs in south China since 2010

| Accession number | Strain name | Accession number | Strain name |
| --- | --- | --- | --- |
| KM374056 | pigeon/Yunnan/1111/2013 | MK469969 | Pi/CH/GXG2/2012 |
| KM374059 | pigeon/Guangxi/1015/2013 | MK469970 | Pi/CH/GXG3/2012 |
| KT381592 | Pigeon/Guangdong/GZ293/2014 | MK469971 | Pi/CH/GXG6/2013 |
| KT381595 | Pigeon/Guangdong/GM1/2014 | MK469972 | Pi/CH/GXG7/2014 |
| KT381601 | Pigeon/Guangdong/GZ287/2013 | MK469973 | Pi/CH/GXG13/2014 |
| KT381602 | Pigeon/Guangdong/GZ288/2013 | MK469974 | Pi/CH/GXG16/2013 |
| KT381603 | Pigeon/Guangdong/GZ290/2013 | MK749297 | Pi/CH/GXG1/2012 |
| KT381604 | Pigeon/Guangdong/GZ292/2014 | MK749298 | Pi/CH/GXG22/2015 |
| KT381606 | Pigeon/Guangdong/GZ289/2014 | MK749299 | Pi/CH/GXG24/2015 |
| KY788663 | Grey_heron/Ch/GD/GZ333/2015 | MK749300 | Pi/CH/GXG25/2015 |
| KY788667 | European_turtledove/Ch/GD/GZ23/2015 | MK749301 | Pi/CH/GXG28/2016 |
| MF580815 | pi/CH/GX0094/2011 | MK749302 | Pi/CH/GXG31/2016 |
| MF580816 | pi/CH/GX0001/2012 | MN477454 | Pi/CH/GXG44/2018 |
| MF580817 | pi/CH/GX0012/2012 | MN632518 | pigeon/Taiwan/AHRI43/2010 |
| MF580818 | pi/CH/GX0019/2012 | MN632519 | pigeon/Taiwan/AHRI68/2012 |
| MF580819 | pi/CH/GX0022/2012 | MN632520 | pigeon/Taiwan/AHRI107/2016 |
| MF580820 | pi/CH/GX0029/2012 | MN632521 | pigeon/Taiwan/AHRI111/2017 |
| MF580821 | pi/CH/GX0031/2012 | MN632537 | dove/Taiwan/AHRI113/2017 |
| MF580822 | pi/CH/GX0034/2012 | MN632538 | pigeon/Taiwan/AHRI114/2017 |
| MF580823 | pi/CH/GX0505/2012 | MN632539 | dove/Taiwan/AHRI115/2017 |
| MF580824 | pi/CH/GX0004/2013 | MN632540 | dove/Taiwan/AHRI116/2017 |
| MF580825 | pi/CH/GX0119/2015 | MN632541 | dove/Taiwan/AHRI117/2017 |
| MF580826 | pi/CH/GX0126/2015 | MN632542 | dove/Taiwan/AHRI118/2017 |
| MF580827 | pi/CH/GX1103/2015 | MN632543 | dove/Taiwan/AHRI120/2017 |
| MG840653 | pigeon/Guizhou/1050/2017 | MN632544 | pigeon/Taiwan/AHRI121/2017 |
| MG840658 | pigeon/Yunnan/1205/2015 | MN632545 | dove/Taiwan/AHRI123/2017 |
| MG840659 | pigeon/Yunnan/1336/2015 | MN632546 | magpies/Taiwan/AHRI125/2017 |
| MG840660 | pigeon/Yunnan/1453/2017 | MN632547 | dove/Taiwan/AHRI126/2017 |
| MK469964 | Pi/CH/GXG20/2015 | MN632548 | pigeon/Taiwan/AHRI133/2018 |
| MK469965 | Pi/CH/GXG29/2016 | MN893303 | pigeon/Guangdong/GZ08/2017 |
| MK469966 | Pi/CH/GXG6/2015 | MN893304 | pigeon/Guangdong/SZ12/2018 |
| MK469967 | Pi/CH/GXG33/2016 | OQ144646 | pigeon/China/Yunnan/1078/2022 |
| MK469968 | Pi/CH/GXG35/2017 | OQ144648 | pigeon/China/Guangxi/1228/2022 |
